# Crustacean Zooplankton Ingestion of Potentially Toxic *Microcystis*: In Situ Estimation Using *mcyE* Gene Gut Content Detection in a Large Temperate Eutrophic Lake

**DOI:** 10.3390/toxins17010042

**Published:** 2025-01-16

**Authors:** Helen Agasild, Margarita Esmeralda Gonzales Ferraz, Madli Saat, Priit Zingel, Kai Piirsoo, Kätlin Blank, Veljo Kisand, Tiina Nõges, Kristel Panksep

**Affiliations:** 1 Institute of Agricultural and Environmental Sciences, Estonian University of Life Sciences, 51006 Tartu, Estonia; margarita.gonzales@emu.ee (M.E.G.F.); madli.saat@emu.ee (M.S.); priit.zingel@emu.ee (P.Z.); kai.piirsoo@emu.ee (K.P.); katlin.blank@emu.ee (K.B.); veljo.kisand@ut.ee (V.K.); tiina.noges@emu.ee (T.N.); kristel.panksep@ut.ee (K.P.); 2 Institute of Technology, University of Tartu, 50411 Tartu, Estonia

**Keywords:** *Microcystis*, toxic cyanobacteria, cladocerans, copepods, aquatic food web, qPCR

## Abstract

Grazing by zooplankton can regulate bloom-forming cyanobacteria but can also transfer toxin-producing cells, as well as toxic metabolites, to the food web. While laboratory investigations have provided extensive knowledge on zooplankton and toxic cyanobacteria interactions, information on zooplankton feeding on toxin-producing cyanobacteria in natural water bodies remains scarce. In this study, we quantified *Microcystis*-specific *mcyE* synthase genes from the gut contents of various cladoceran and copepod taxa to assess the in situ crustacean community and taxon-specific ingestion of potentially toxic *Microcystis* in Lake Peipsi, a large eutrophic lake in Estonia, Northern Europe. *Microcystis* cells with *mcyE* genes were found in all crustaceans examined. However, some species, such as the cyclopoid copepod *Mesocyclops leuckarti*, were more efficient in ingesting potentially toxic *Microcystis* than other co-occurring cladocerans (*Daphnia* spp., *Bosmina* spp., *Chydorus sphaericus*) and copepods (*Eudiaptomus gracilis*). The amount of toxigenic *Microcystis* cells grazed by crustacean population changed temporarily, and copepods were the predominant consumers of toxigenic *Microcystis* during several months of the 5-month study period. Crustacean ingestion of toxigenic *Microcystis* was not related to *Microcystis* biomass or *mcyE* gene copy numbers in the environment but was instead related to the abundance of major crustacean grazers. Our findings emphasize the close interaction between crustacean zooplankton and toxigenic *Microcystis*, indicating that some species may play a more significant role in linking toxic cells within the food web than others.

## 1. Introduction

Cyanobacterial blooms and their metabolites are a significant global concern, impacting freshwater ecosystems, fisheries, and tourism [1,2,3]. In aquatic food webs, zooplankton play a crucial role in regulating algal biomass, also feeding on potentially toxic cyanobacteria such as *Microcystis*. Since most microcystins are intracellularly bound within cyanobacterial cells [4], zooplankton feeding on these toxigenic cells becomes a significant mechanism for removing toxic algae from the water. Zooplankton collect the toxigenic cells in their guts and store the assimilated toxins in their tissues [5,6]. This, however, highlights the contradictory role of zooplankton grazing in the aquatic ecosystem. While the consumption of toxigenic cyanobacterial cells reduces harmful blooms and lowers cyanotoxin risk in the environment, zooplankton also serve as food for juvenile and planktivorous fish. Thus, they form a key link in transferring ingested toxic cells and accumulating intracellular microcystins up the food chain [7].

The highest risk from toxic cyanobacteria in ecosystems, impacting food webs and fish, is typically associated with the peak occurrence of toxin-producing strains or toxic metabolites in the environment [8]. Cyanotoxins, such as microcystins produced by *Microcystis* spp., can accumulate in aquatic organisms, leading to bioaccumulation through the food web. This can result in significant health risks for higher trophic levels, including fish, birds, and mammals, as reviewed in Chen et al. [9,10]. However, when considering zooplankton as the primary vector between cyanotoxins and fish in water bodies, the dynamics of major grazing zooplankton and the production of toxic strains may not always align. The consumption of toxigenic cells by zooplankton and the subsequent accumulation of cyanotoxins depend not only on the abundance of microcystin-producing strains in the environment but also on the presence of grazers capable of feeding on toxic strains [5]. For instance, general grazing zooplankton, such as *Daphnia*, may ingest a large number of potentially toxic cells [11] before being preyed upon and digested by predator organisms [7,12]. Although some metazoan zooplankters, such as small bacterivorous rotifers and cyclopoid copepods, may be abundant during bloom periods, larger-sized crustacean species (e.g., daphniids, calanoids) tend to decline in many eutrophic lakes during periods of massive development of potentially toxic cyanobacteria [13,14].

Several factors may contribute to this phenomenon, such as the adverse effects of cyanotoxins directly impacting zooplankton grazing rates, abundance, and behavior [15,16,17]. Poor food conditions due to low content levels of essential biomolecules affecting reproduction, including interference from filamentous cyanobacteria during feeding, also play a role [18,19]. Additionally, intensive predation by newly hatched 0+ fish can alter grazers’ abundance and community structure during the summer bloom [20].

In temperate lakes, the onset of blooms typically coincides with favorable light and temperature conditions, leading to peak biomass during the warmest months [21,22]. In freshwater ecosystems, microcystin production is most frequently documented from the genera *Microcystis*, *Dolichospermum*, and *Planktothrix*, and species from *Microcystis* are the predominant producers of microcystins in eutrophic waters worldwide [23,24]. The growth rate of the genus *Microcystis* is highly sensitive to water temperature, accelerating at temperatures above 10 °C [25,26]. Similarly, zooplankton abundance and biomass increase when temperature and food availability support active production and population growth [27]. Toxic and non-toxic strains of the same cyanobacterial species often coexist in the environment [24,28]. Environmental factors, including nutrient concentrations, temperature, and pH, can influence their relative abundance [29,30,31,32] and chemical interactions with grazers [33]. This highlights the complexity of interactions affecting the timing of the flow of toxic cyanobacteria into the food web. There remains an open question of whether the transfer of toxic cells to pelagic food web is primarily driven by the dynamics of major grazers or by the abundance of toxigenic strains in the water. To our knowledge, no studies have directly addressed this in natural water bodies yet.

PCR methods have emerged as promising tools for the in situ estimation and quantification of zooplankton ingestion of toxic cyanobacteria. For instance, research demonstrated that the copepod species *Pseudodiaptomus forbesi* can ingest toxic *Microcystis* strains during blooms in the San Francisco Estuary [5]. Both microzooplankton (20–200 µm) and mesozooplankton (200–2000 µm) communities have been observed to feed on both toxic and non-toxic strains of *Microcystis* [34]. Furthermore, Sotton et al. [7] demonstrated the transfer of toxic *Planktothrix rubescens* via grazing zooplankton to predatory zooplankton, which mediated the microcystin contamination in zooplanktivorous whitefish. Although qPCR detection of toxin-producing genes from consumers is rarely applied in field investigations, it could provide valuable information regarding toxic cyanobacteria–food web interactions. Specifically, it could shed light on major trophic links involved in the movement of cyanotoxins and contribute to understanding the regulation of toxigenic cyanobacteria in natural environments.

**Figure 1:**
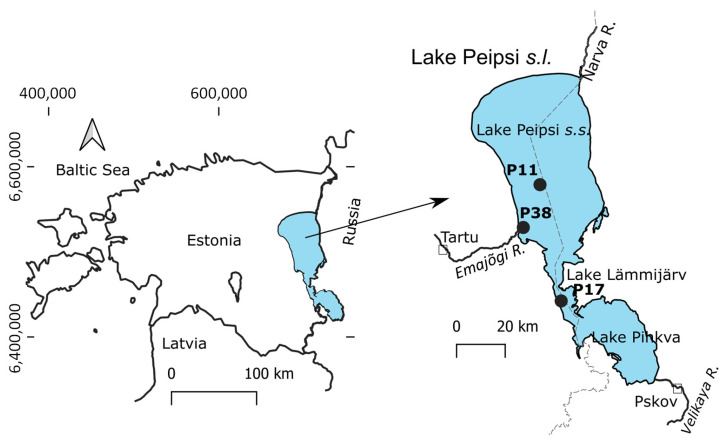
Location of sampling sites in Peipsi *sensu lato* (*s.l.*): P11 and P38 in Peipsi *sensu stricto* (*s.s.*) and P17 in Lämmijärv.

To address the knowledge gap, we studied the taxon-specific ingestion of potentially toxic *Microcystis* cells by major cladoceran and copepod taxa among crustacean zooplankton in the large, shallow Lake Peipsi (Figure 1). We focused on toxigenic *Microcystis* as the major bloom-forming cyanobacteria in this lake. In Peipsi, *Dolichospermum* spp. and *Planktothrix* spp. can also produce microcystins, but the respective *mcyE*-producing cells are present in considerably lower abundances compared to the concurrent *Microcystis* spp. [35]. Previous analyses of the ingestion of potentially microcystin-producing *Dolichospermum* and *Planktothrix* by different zooplankton species have not detected the ingestion of these cyanobacteria above the rate of the reliable quantification level [11]. Therefore, we presume that *Microcystis* is the major microcystin-producing cyanobacteria moving through the pelagic food web in Peipsi.

Our previous research revealed that the major crustaceans—*Daphnia* spp., *Bosmina* spp., *Eudiaptomus gracilis*, and the predatory cladoceran *Bythotrephes longimanus*—can ingest cyanobacteria, including potentially toxic *Microcystis*, either directly or indirectly [11]. However, the research indicated that some species might be more efficient at this process and thus could potentially be more significant mediators of toxic cyanobacteria to the aquatic food web.

Our working hypotheses were that (1) despite different feeding modes, various taxa in the crustacean community are able to ingest toxic *Microcystis* cells; (2) cladocerans, specifically the generalist feeder *Daphnia*, form the central link connecting toxigenic *Microcystis* to the food web in a eutrophic lake; and (3) the ingestion of toxigenic *Microcystis* by the crustacean community depends more on the dynamics of efficient consumers of toxic cells than on the seasonal availability of toxin-producing *Microcystis* in the environment.

## 2. Results

### 2.1. Environmental Variables

During the study period, the total phosphorus (TP), total nitrogen (TN), and chlorophyll-*a* (Chl-*a*) values increased from the northern basin towards the central basin (Lämmijärv) (Table 1). Water temperature was relatively similar at all three study sites, peaking in July at a maximum of 25.7 °C. Based on water quality parameters and the OECD classification [36], Lake Peipsi *sensu stricto* (*s.s.*) is classified as eutrophic, while Lämmijärv is considered a hypertrophic part of the lake.

### 2.2. Seasonal Dynamics of Phytoplankton, Microcystis spp. And mcyE Copy Numbers

**Figure 2:**
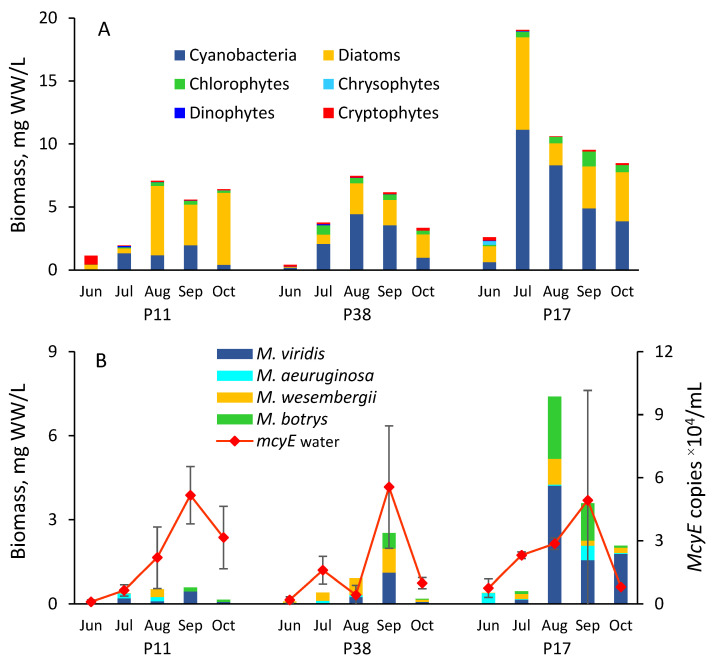
Phytoplankton composition and biomass (**A**); seasonal dynamics of biomasses of *Microcystis* species and *Microcystis mcyE* copy numbers (±SD) (**B**) in sampling sites P11, P38, and P17 in Lake Peipsi in 2021.

Cyanobacteria and diatoms dominated the phytoplankton biomass (Figure 2A). Cyanobacterial biomass varied between 0.04 and 11.14 mgWW/L across the sampled areas and periods. *Microcystis* formed 4 to 89% of the cyanobacterial biomass and was presented by species *M. viridis*, *M. aeruginosa*, *M. botrys*, and *M. wesenbergii*. The biomass of *M. viridis* was particularly notable, forming the highest biomass in all sampling sites, 12.8–26.8% of total cyanobacteria, followed by *M. wesembergii* (5.1–16.7%), *M. botrys* (14.5–12.9%), and *M. aeruginosa* (2–6.7%). *Microcystis* peaked in August (P17) or in September (P11, P38), with approximately 2- to 4-times higher biomasses in the hypertrophic part of the lake (P17) compared to P11 and P38 in Peipsi *s.s.* (Figure 2B). Despite these biomass differences, the abundances of *Microcystis* with *mcyE* synthetase genes were generally similar in all sites, with peak occurrences in September (Figure 2B).

### 2.3. Seasonal Dynamics of Crustacean Zooplankton Communities

**Figure 3:**
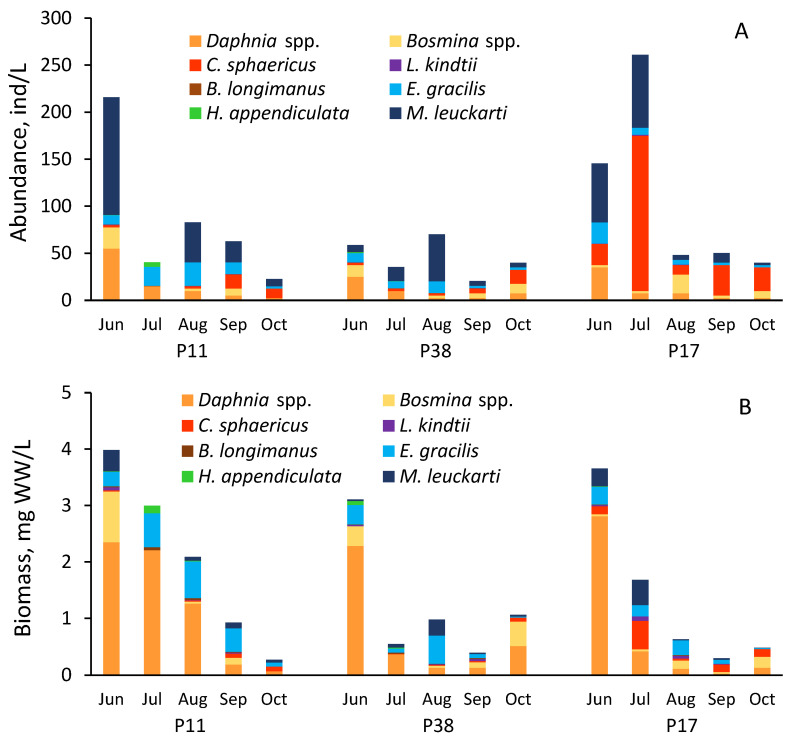
Seasonal dynamics of abundance (**A**) and biomass (**B**) of major crustacean taxa in sampling sites P11, P38, and P17 in Lake Peipsi in 2021.

The abundance and biomass of the studied crustaceans (cladocerans, adults copepods, and copepodites) ranged from 20 to 803 individuals/L and 0.27–3.98 mgWW/L, respectively (Figure 3), comprising an average of 90% of the whole crustacean biomass (composition, abundance, and biomass shown in Supplementary Figure S1). *Daphnia* spp. (*D. galeata*, *D. longispina*, *D. cristata*, and *D. cucullata*) among filtering cladocerans, and *Eudiaptomus gracilis* and *Mesocyclops leuckarti* among copepods were the most abundant taxa in the studied lake areas. Crustacean abundance, biomass, and composition varied across the different lake areas. Larger-sized species, such as *D. galeata* and *E. gracilis*, were generally most abundant in the eutrophic Peipsi *s.s.* (P11). By contrast, smaller-sized species like *M. leuckarti* and *Chydorus sphaericus* dominated the population in the hypertrophic part of the lake (Lämmijärv, P17). Predatory cladocerans *Leptodora kindtii* and *Bhytothrephes longimanus*, as well as the large current-feeding copepod *Heterocope appendiculata*, were presented in limited numbers.

### 2.4. Crustacean Ingestion of Potentially Toxic Microcystis

*Microcystis mcyE* gene copies were detected in the guts of all analyzed crustacean taxa. Taxon-specific ingestion of *Microcystis mcyE*-containing cells varied across dates and sites (Supplementary Figure S2). The highest taxon-specific ingestion was measured for the current-feeding copepod *H. appendiculata* and the ambush-feeding copepod *M. leuckarti* (Table 2). Among filter-feeding cladocerans, *Daphnia* spp. exhibited higher values than *Bosmina* spp. and *C. sphaericus*. Throughout the analyzed period, *Microcystis* cells with *mcyE* genes were most frequently detected in the guts of *M. leuckarti* and *Daphnia* spp., while in predatory cladocerans (*L. kindtii*, *B. longimanus*), this was detected only sporadically.

Maximum ingestion rates indicated that *Daphnia* spp. was capable of ingesting hundreds of *Microcystis* toxigenic cells (303 *mcyE*-containing cells/ind), whereas smaller-sized cladocerans, *Bosmina* spp. and *C. sphaericus*, had fewer toxigenic cells in their guts. Among copepods, *H. appendiculata* had the highest number of *mcyE*-containing cells (6429 cells/ind), followed by *M. leuckarti*.

Weight-specific ingestion of potential microcystin-producing cells was, on average, highest in *H. appendiculata* (611 *mcyE*-containing cells/100 mg of grazer body mass) and *M. leuckarti* (494 *mcyE*-containing cells/100 mg) (Table 2). Cladocerans had much lower weight-specific ingestion, with the highest average values estimated for the smallest cladoceran, *C. sphaericus* (216 *mcyE*-containing cells/100 mg) (Table 2).

**Figure 4:**
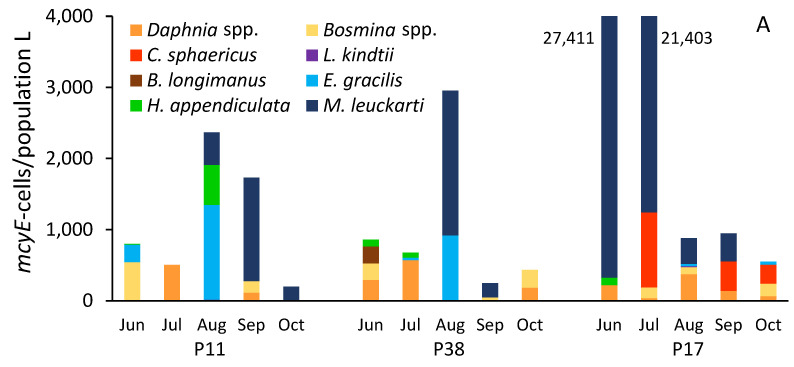

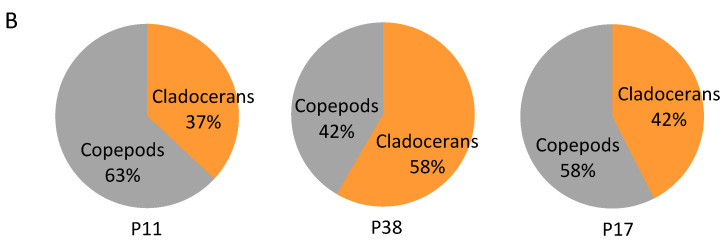
Seasonal dynamics of crustacean population feeding on potentially toxic *Microcystis* cells (based on the detection of *mcyE*-containing cells in consumer’ guts) in sampling sites P11, P38, and P17 in Lake Peipsi in 2021; ingestion by various cladoceran and copepod taxa (**A**); proportional contribution (%) of cladoceran and copepod ingestion (**B**).

Total crustacean population feeding (sum of cladoceran and copepod ingestion) on *Microcystis* toxigenic cells varied between 199 and 27,736 *mcyE*-containing cells/population L, with most dates showing values below 1000 *mcyE* cells/population L. Crustacean ingestion levels were similar in P11 and P38 but remarkably higher in June and July in P17 (Figure 4A; Supplementary Figure S3). On average, across the sampling areas and period, the copepod community (*M. leuckarti*, *E. gracilis*, *H. appendiculata*) was slightly more efficient in ingesting the microcystin-producing cells, accounting for 54% compared to 46% for cladocerans (*Daphnia* spp., *Bosmina* spp., *C. sphaericus*, *L. kindtii*, *B. longimanus*) (Figure 4B). When present in plankton, the cyclopoid copepod *M. leuckarti* often constituted the majority of crustacean ingestion of *Microcystis* microcystin-producing cells, particularly notably in P17 during June and July. Additionally, *Daphnia* spp., *C. sphaericus*, and *E. gracilis* made significant contributions on certain dates and sites (Figure 4A).

### 2.5. Relationships of Crustacean Ingestion of mcyE-Containing Cells with Crustacean Assemblage Composition, Microcystis Biomass, and Environmental Parameters

First, we tested the sampling site difference in *mcyE* abundances and crustacean populations’ ingestion (cladocerans, copepods, total crustaceans). As there were no statistical differences in *mcyE* abundance and grazer population ingestion of potentially toxic *Microcystis* cells between the lake sites (Mann–Whitney U-test, multiple pairwise comparisons, *p* > 0.05), we pooled the data from all three sites and analyzed lake-wide relationships of crustacean ingestion of *mcyE*-containing cells between crustacean assemblage and environmental parameters.

Secondly, in analyzing the relationship between crustacean ingestion and variable grazers’ abundances and biomasses, we found a significant positive correlation between the ingestion of potentially toxic *Microcystis* cells by the total crustacean population and copepod abundances (r = 0.68, *p* < 0.01) (Table 3). Among the copepod species analyzed, the strongest relationship was found with the abundances and biomasses of *M. leuckarti* and *E. gracilis*. Respective correlations were not observed for cladocerans. The biomass of *Daphnia* spp. and the biomasses of cladocerans and crustaceans were significantly negatively correlated to the biomass of *Microcystis* and *mcyE* gene copy numbers in lake water. The ingestion of *mcyE*-containing cells (by cladocerans, copepods, total crustaceans, or individual crustacean taxa) was not significantly correlated with *Microcystis* biomass or the *Microcystis mcyE* copy numbers in lake water (Spearman correlations, *p* > 0.05).

**Figure 5:**
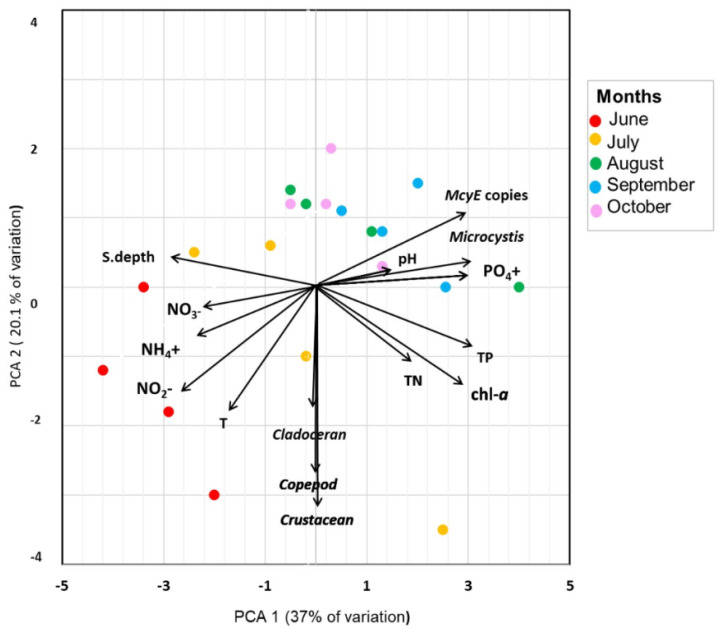
Principal component analysis plot displaying the association between cladoceran, copepod, and total crustacean population ingestion (*mcyE* cell/L), *Microcystis* biomass, *McyE* copy numbers, and environmental variables in Lake Peipsi in 2021 grouped by month.

Finally, we tested the relationships between cladoceran, copepod, and total crustacean ingestion of toxigenic *Microcystis* and environmental physio-chemical parameters. Spearman correlation analyses showed clear positive relationships with nutrients, *Microcystis* biomass, and *mcyE* gene copy numbers in lake water but no significant relationships with crustacean population (cladoceran, copepod, or total crustacean) ingestion of potentially toxic *Microcystis* (Table 4). Similarly, no association between environmental parameters and cladoceran, copepod, and total crustacean population ingestion of toxigenic *Microcystis* cells was observed from the principal component analysis (PCA) across the studied months (Figure 5). The PCA shows cladoceran, copepod, and total crustacean population ingestion, *Microcystis* species biomasses, and *Microcystis mcyE* copy numbers as variables, linearly fitting the environmental variables of nutrients (TP, PO_4_^+^, TN, NO_3_^−^, NO_2_^−^, NO_4_^+^), temperature, pH, Secchi depth, and chl-*a* with the PCA ordination space. The first component (PCA1) and second component (PCA2) combined accounted for 57.1% of the variation (PCA1 = 37%; PCA2 = 20.1%).

## 3. Discussion

Laboratory analyses have demonstrated that various zooplankton taxa are capable of consuming toxigenic *Microcystis* [37]. We confirmed this in situ in a eutrophic lake, showing that despite different feeding modes, all analyzed cladoceran and copepod taxa (*Daphnia* spp., *Bosmina* spp., *C. sphaericus*, *M. leuckarti*, *E. gracilis*, *H. appendiculata*, *L. kindtii*, *B. longimanus*) contained toxin-producing *Microcystis* cells in their guts. Most of these taxa (e.g., *Daphnia* spp., *Bosmina* spp., *C. sphaericus*, *M. leuckarti*, and *E. gracilis*) are commonly occurring among zooplankton communities in eutrophic waters [38]. They could thus impact *Microcystis* populations in these waterbodies. In Peipsi, these taxa constitute a major part of zooplankton biomass [39].

In this study, the feeding assessment of various taxa provided an interesting insight into crustacean zooplankton interactions with toxigenic *Microcystis*.

We expected that the generalist-feeding *Daphnia* that is able to feed on variable types and sizes of algae, including toxic cyanobacteria [37], has the highest capability to collect *Microcystis* toxigenic cells among the studied crustaceans. However, this expectation was not confirmed. Although *Daphnia* spp. had the highest taxon-specific ingestion rate among filtering cladocerans, and it showed the capability to ingest microcystin-producing cells throughout the season (as seen in hypertrophic P17), surprisingly, copepods were generally more efficient in collecting the toxigenic *Microcystis*. This made copepods the predominant feeders of toxigenic *Microcystis* for several months in Peipsi, while cladocerans contributed earlier in the season when daphniids were abundant in the plankton.

We found significant interspecific differences in the consumption of the toxigenic *Microcystis*, especially among copepods. Although occurring in low numbers in Peipsi, the large-sized calanoid *H. appendiculata* was able to consume toxigenic cells with high taxon-specific ingestion. A relatively high number of toxigenic cells was also detected in the guts of the small cyclopoid copepod *M. leuckarti*. It is important to note that, due to their generally omnivorous nature of feeding [40,41,42], copepods may obtain toxic cyanobacterial cells either by feeding directly on algae or through their animal prey, such as rotifers and protozoa [34]. This may explain the substantial numbers of potentially toxic *Microcystis* cells in the guts of *M. leuckarti* and *H. appendiculata* compared to cladocerans.

However, the opposite results were obtained for another calanoid copepod, *E. gracilis*. Laboratory experiments revealed that this species can successfully avoid consuming toxic strains through cue-based selective avoidance triggered by cellular microcystin and can efficiently graze on alternative, non-toxic prey [15,43]. Such behavior could also explain the limited ingestion of toxigenic *Microcystis* by *E. gracilis* in Peipsi. Analyzing all sites and dates of this study, *E. gracilis* had generally fewer samples, indicating ingestion of toxic *Microcystis* than other co-occurring crustaceans. An earlier in situ zooplankton feeding study from Peipsi, which assessed gut phytoplankton marker pigment composition [11], indicated an active selection against colonial and filamentous cyanobacteria but a high preference for cryptophytes in this copepod. Collectively, these results indicate that *E. gracilis* has a modest effect on toxic *Microcystis* in Peipsi and probably in other lakes with high *Microcystis* biomass.

Analyzing of crustacean weight-specific ingestion revealed that the small-sized crustaceans *C. sphaericus* and *M. leuckarti*, which are characteristic species of highly eutrophic water bodies [27,44,45], are capable of high weight-specific ingestion of potentially toxic *Microcystis*. Based on crustacean population ingestion, we can presume that, with abundant populations, as observed in hypertrophic P17 in this study, *C. sphaericus* and *M. leuckarti* are capable of removing a substantial amount of toxigenic cells from the lake water. The observed pattern may indicate a high tolerance or effective detoxification of microcystins [46], which may explain their prevalence in bloom-dominated lakes. However, future research is necessary to confirm this. Research from hypertrophic Lake Ringsjön (Sweden) has shown that a natural community dominated by cyclopoid copepods and small cladocerans could suppress the blooms of potentially toxic *Dolichospermum*, *Microcystis*, and *Planktothrix* species [47]. This study did not assess the grazing impact; still, as toxic and non-toxic strains of *Microcystis* co-occur in the environment [28], and the tested zooplankters are likely able to ingest both [34,48,49], it is assumed that the total crustacean feeding on *Microcystis* was much higher than the measured ingestion of only toxigenic strains in this study.

Although this was not the scope of our study, we presumed that *Microcystis* colonies were ingested either directly, as smaller and medium-sized (<60 μm) colonies can be consumed by various crustaceans [50,51,52], or alternatively via scraping the surficial cells of larger colonies [53,54]. The colony size of *Microcystis* typically increases with the development of cyanobacterial blooms [55], making them easier for grazers to handle earlier in the season [56]. In Peipsi in July 2014, the colony sizes of *Microcystis* spp. ranged from 48 to 96 μm in diameter, with a predominance of smaller colonies; by September, the share of larger colonies had increased (Kersti Kangro, Estonian University of Life Sciences, unpublished data). The use of colonial cyanobacteria, such as *Microcystis*, as a food along with other algae aligns with our earlier crustacean zooplankton feeding study in Peipsi [11]. Gut phytoplankton marker pigment analyses indicated that colonial cyanobacteria (characterized by the carotenoids zeaxanthin and canthaxanthin) often constituted significant proportions and were preferred algae in the diets of *Daphnia* spp. and *Bosmina* spp. At the same time, diatoms, chlorophytes, and cryophytes were less prevalent. Other crustaceans, such as *E. gracilis*, which preferentially feed on cryptophytes, likely cannot completely avoid ingesting *Microcystis*.

A major result of our study is that the potential risk of toxic cyanobacteria to the aquatic food web and ecosystem cannot be solely assessed based on the dynamics of the abundance of toxigenic strains in the environment despite the common presumptions [8]. As zooplankton represent the primary link transferring the toxic *Microcystis* into the food web, their role in this process must be thoroughly assessed. In Peipsi, the crustacean community feeding on toxigenic strains was inversely related to *Microcystis* biomass and the *mcyE* gene abundance in lake water. Instead, feeding was positively correlated with the abundance of copepods, primarily of the small cyclopoid *M. leuckarti*. This supports the view that copepods might play a significant role in consuming the toxic *Microcystis* in Peipsi. Thus, the occurrence and abundance of major consumers seem to be the main factor determining the grazing dynamics of toxic cells. This is further illustrated by the fact that in all studied areas of Peipsi, the dynamics of the biomasses of crustaceans, particularly cladocerans, and *Microcystis*, with its toxin-producing strains, had opposite patterns. These results highlight the predominant role of grazers, rather than toxin-producing strains, in controlling the flow of toxic *Microcystis* into the pelagic food web.

The widespread presence of toxic *Microcystis* cells in the diet of various crustacean zooplankton observed in this study provides further insights into the functioning of food webs in lakes dominated by toxic bloom-forming *Microcystis*. Based on analyses in 2021 in Peipsi, our results suggest that during the growing period, the toxic cyanobacteria were actively incorporated into the pelagic food web as the lake water consistently contained toxigenic *Microcystis*-contaminated zooplankton. This also poses a potential toxicity threat to young-of-the-year fish, as well as zooplanktivorous smelt (*Osmerus eperlanus* morfa *spirinchus)* and vendace *(Coregonus albula*), which primarily rely on crustacean zooplankton [57,58]. Microcystin concentrations, however, have not yet been measured in fish or zooplankton from Lake Peipsi.

*Microcystis* species common in our lake are all documented for their ability to produce microcystins, although their toxicity levels vary. While *M. aeruginosa* is a well-known microcystin producer, other species, such as *M. wesenbergii*, *M. viridis*, and *M. botrys*, also contribute to the microcystin burden in the environment [1,59]. Based on a previous study in Peipsi, LC-MS/MS analysis identified a total of 14 microcystin variants in the samples, with MC-RR being the most abundant, found in 93% of analyzed samples, followed by MC-LR and its methylated variants in 92% of the samples. These microcystin variants were closely associated with *M. wesenbergii* and *M. aeruginosa* [35]. Available data of microcystin concentrations in the lake have indicated relatively low concentrations (<1 mg/L) of microcystins in the depth-integrated water samples in open water areas but extremely high concentrations (>2000 μg/L) in inshore scum areas [35,60].

## 4. Conclusions

Our research provided a unique example from a natural lake, indicating that various crustacean zooplankters are capable of consuming and transferring toxigenic *Microcystis* to the pelagic food web. We also showed that some crustacean species (e.g., *M. leuckarti* in Lake Peipsi) are more efficient at collecting toxic cells from the environment than other co-occurring species (e.g., *E. gracilis*). Our findings further emphasize that relying solely on *mcyE* gene dynamics in water may introduce bias when assessing the temporal *Microcystis* toxicity risk to the food web. Understanding the dynamics of major grazers may be beneficial for predicting the temporal flow of toxigenic cells to zooplankton and zooplanktivorous fish. However, it must be noted that this current study is based on only one-year analyses of grazer feeding activity on potentially toxic *Microcystis*. The dynamics of toxin-producing strains and zooplankton communities may substantially vary between the years within the same waterbody [61,62] and alter the grazing patterns. We, therefore, encourage further research to verify the results of this study to obtain better predictions of food web contamination with zooplankton-grazed toxic cells and assimilated cyanotoxins in lakes with *Microcystis* bloom occurrences.

## 5. Materials and Methods

### 5.1. Study Site

Lake Peipsi *s.l.* (*sensu lato*) (57°51′–59°01′ N, 26°57′–28°10′ E, 30 m a.s.l.) is a large non-stratified eutrophic lake between Estonia and Russia with a surface area of 3555 km^2^ and mean and maximum depth of 7.1 m and 15.3 m, respectively (Figure 1). Lake Peipsi *s.l.* consists of three basins from north to south: Peipsi *s.s.*, Lämmijärv, and Pihkva. Due to the large area, variable hydrology, morphometry, and bottom topography, the lake varies in its trophic state, with increasing total phosphorus (TP), total nitrogen (TN), and chlorophyll-*a* (chl-*a*) values from the northern basin (Peipsi *s.s.*) towards the central basin (Lämmijärv) and southern basin (Pihkva) [35,63]. During the growing season (May–October), diatoms and cyanobacteria prevail in the phytoplankton biomass. *Microcystis* is the major toxin-producing cyanobacterial genus, and, along with other potentially toxic genera such as *Gloeotrichia*, *Dolichospermium*, *Aphanizomenon*, and *Planktothrix*, forms the highest cyanobacterial biomass during the summer months (July–August) or early autumn (September) [35]. Cyanobacterial biomass, including *Microcystis*, increases towards the southern basins. Conversely, metazoan zooplankton have higher biomass values in the northern, moderately eutrophic Peipsi *s.s.* (mainly formed by *Daphnia galeata* and *Eudiaptomus gracilis*) compared to the hypertrophic Lämmijärv, where generally smaller species prevail (*Daphnia cucullata*, *Chydorus sphaericus*, *Mesocyclops leuckarti*) [64]. The lake is typically ice-covered from December to April [65]. The water is well mixed by wind and well aerated by waves and currents, with no permanent stratification of temperature, dissolved oxygen, or hydrochemical parameters during the ice-free period.

### 5.2. Sampling

In this study, samples were collected monthly from June to October 2021 from the routine state monitoring stations in Lake Peipsi (*s.s.*) (P11, P38) and Lämmijärv (P17) (Figure 1). Water chemistry analyses were performed as part of the state monitoring program by the Estonian Environmental Research Centre following international and Estonian quality standards.

Depth-integrated water at 1 m intervals was collected with a Limnos water sampler (with a free flow design for vertical sampling) from the entire water column and mixed in a tank. Subsamples were taken from this water for analyses of phytoplankton community composition and biomass, molecular detection of *mcyE* genes of potentially toxic *Microcystis*, and zooplankton community composition, abundance, and biomass.

To identify zooplankton composition and biomass, 20 L of the depth-integrated water was filtered through a 48-μm mesh plankton net and concentrated into a 200 mL sample jar. Phytoplankton and zooplankton samples were fixed with acidified Lugol’s solution at a final concentration of 1% and kept in the dark until further analysis.

To assess the abundance of *mcyE* genes in water samples, 90 to 1000 mL of the depth-integrated water was filtered on-site using Sterivex filter capsules (pore size 0.2 µm) (Merck Millipore Sterivex™, Darmstadt, Germany). Samples were filtered in triplicate. Filters were stored in 96% ethanol at −80 °C until further analysis.

For analyzing potentially toxic *Microcystis* in crustacean gut content, depth-integrated samples were collected with vertical tows of a plankton net (300 μm mesh) until a sufficient amount of material was obtained. The collected bulk zooplankton was instantly rinsed with deionized water to clean the sample from phytoplankton as much as possible, concentrated in a small volume, and immediately frozen in liquid nitrogen. The samples were preserved at −80 °C in the laboratory until further analyses.

### 5.3. Phyto- and Zooplankton Biomass, Crustacean Preparation for Molecular Analyses

Phytoplankton cells were enumerated with an inverted microscope (Nikon Eclipse Ti-S, Nicon Instruments INC, Melville, NY, USA) at ×400 magnification, using the Utermöhl technique [66]. Phytoplankton taxa were identified to the lowest possible taxonomic level, and each counted taxon was converted to biovolumes by measuring cell, trichome, or colony dimensions. Phytoplankton biomass was expressed as mg WW/L (milligrams of wet weight per liter of lake water).

Zooplankton biomass and community composition were analyzed under a stereomicroscope (Nikon SMZ1500, Nicon Instruments INC, Melville, NY, USA, up to ×120 magnification) in a Bogorov chamber. Crustacean length was converted to wet weight as described by Studenikina and Cherepakhina [67] and Balushkina and Winberg [68]. Zooplankton taxa accounting for 20% or more of the biomass were considered abundance and biomass dominants, respectively [69].

To prepare zooplankton samples for molecular analyses, the frozen bulk zooplankton samples were thawed to separate the most abundant taxa. These consisted of three dominant grazing cladocerans, *Daphnia* spp. (*D. galeata*, *D. longispina*, *D. cristata*, *D. cucullata*), *Bosmina* spp. (*B. berolinensis*, *B. gibbera*, *B. thersites*), and *Chydorus sphaericus*; two current-feeding calanoid copepods, *E. gracilis* and *Heterocope appendiculata*; one ambush-feeding cyclopoid copepod, *M. leuckarti*; and two predatory cladoceran species, *Leptodora kindtii* and *Bhytotrephes longimanus*. Whenever possible, three replicates were made per sample. For samples of *Daphnia* spp. and *E. gracilis*, 50 individuals were collected per replicate whenever possible. For smaller taxa (*Bosmina* spp., *C. sphaericus*, *M. leuckarti*), 50–200 individuals were separated. For larger-sized crustaceans (*H. appendiculata*, *L. kindtii*, and *B. longimanus*), generally fewer than 50 individuals per replicate were separated. For copepod samples, only adult and copepodite stages were used. Lengths of approximately 30 individuals per sample were measured to calculate the weight-specific ingestion of various crustacean grazers. The collected specimens were repeatedly rinsed with deionized water to minimize contamination by non-ingested algae, visually inspected to ensure no external algal cells were stuck on the animals, and then placed into 1.5 mL microtubes for DNA extraction.

### 5.4. DNA Extraction and Molecular Analyses

Genomic DNA from zooplankton was extracted using the NucleoSpin^®^ Tissue Kit (MACHEREY-NAGEL, Düren, Germany) following the manufacturer’s instructions. DNA from integrated water samples was extracted using a modified NucleoSpin^®^ Tissue Kit for Sterivex filter capsules. All extractions were made under the laminar flow hood to protect samples and avoid contamination. The initial steps of the extraction protocol were modified as follows: (1) Ethanol was removed from the SX filter capsule using a 3 mL syringe and centrifuged at 5000 RCF for 30 min. (2) Meanwhile, the SX filter capsules were cut open, and the filter was removed using a sterile scalpel and forceps and placed into 5 mL safe-lock tubes. (3) After centrifugation, the remaining ethanol was carefully removed from the pellet; the pellet was therefore resuspended in 630 µL of warm T1 buffer and 70 µL of Proteinase K. (4) The resuspended pellet in T1 buffer and Proteinase K mixture was pipetted onto the SX filter. (5) Garnet and glass beads (Qiagen, Hilden, Germany) were added to the filters, and the mixture was vortexed at high speed for 10 min. (6) The samples were then incubated at 56 °C overnight to lyse. (7) After the incubation step, the rest of the protocol was followed as instructed in the protocol, but the volume for buffer B3 and ethanol was 700 µL.

The quality and quantity of the extracted DNA were assessed using a NanoDrop 2000 UV-Vis spectrophotometer (Thermo Fisher Scientific Inc., Waltham, MA, USA). The DNA was stored at −80 °C until further analysis. To quantify potential microcystin producers among the genera *Microcystis*, in the samples, *Microcystis*-specific *mcyE* qPCR was performed using an absolute quantification method with an internal standard curve. MC-producing *Microcystis* sp. 205 (HAMBI/UHCC Culture Collection, University of Helsinki) was used to construct standard curves in the qPCR analysis. Information about the approximate genome size of *Microcystis* was taken into account for calculations. A more detailed description about the standard curve construction and calculations can be found in Koskenniemi et al. [70]. Each 10 µL qPCR reaction mixture included the following components: 1× HOT FIREPol^®^ Probe Universal qPCR Mix (Solis BioDyne, Tartu, Estonia), 0.3 µM of both forward and reverse primers, 0.3 µM of a fluorescently labeled TaqMan probe, 5 µL of 10-fold diluted template DNA, and molecular-grade dH_2_O to make up the remaining volume. Each environmental sample was tested in triplicate. Additionally, each qPCR analysis plate contained negative control samples and positive standard DNA dilutions. All qPCR reactions were conducted on a LightCycler^®^ 480 System (Roche Life Science, Indianapolis, IN, USA) using a 384-well platform using the following protocol: 95 °C for 12 min for initial denaturation, 40 cycles of 95 °C for 15 s, and 62 °C. Results were analyzed using LightCycler^®^ Software 1.5. The *mcyE* gene was chosen to detect and quantify potential microcystin-producing *Microcystis* because of its established role in microcystin production and its reliability as a molecular marker. Since *mcyE* is typically found as a single copy per genome, it is ideal for assessing the abundance of potentially toxic *Microcystis* cells in both environmental samples and grazers [31,71,72].

### 5.5. Data Analyses

The abundance of potentially toxic cells in zooplankton guts represents their last feeding activity before sampling. Based on the abundances of *Microcystis* cells with the *mcyE* gene in zooplankton gut contents, we calculated the following indices: taxon-specific ingestion, maximum ingestion, weight-specific ingestion, and population ingestion (cladoceran, copepod, total crustaceans). Taxon-specific ingestion was calculated as the mean value of results of replicated qPCR analysis for each date when the taxon’s sample was prepared; the results are given as ingestion of *mcyE*-containing cells per individual. Maximum ingestion is the highest measured abundance of cells with the *mcyE* gene in consumer gut content and refers to the taxon’s highest capability to ingest potentially toxic *Microcystis* cells under in situ conditions. Weight-specific ingestion was calculated using the taxon’s average weight and ingestion of *Microcystis* cells with the *mcyE* gene, provided as *mcyE*-containing cells per 100 μg of grazer body mass. Population ingestion (cladoceran, copepod, or total crustacean) of *Microcystis* cells with the *mcyE* gene was calculated by summing taxon-specific ingestions, considering the respective consumer numbers in one liter of lake water.

For statistical analyses, the principal component analysis was performed to analyze the ingestion of cells with *mcyE* synthetase genes and the biomass of *Microcystis* and to compare the consumption (population ingestion) by different zooplankton in various months. We also analyzed the effects of environmental variables on zooplankton ingestion and *Microcystis* biomass. PCA was performed with the function “prcomp”. Spearman’s rank correlation (r_s_) with the function “cor.test” was used to determine the relationship between the abundance and biomass of zooplankton, *Microcystis* biomass, *mcyE* abundance, and physiochemical indices versus crustacean ingestion on *mcyE*-containing cells. PCA and Spearman’s rank correlation analyses were performed with the RStudio 4.1.2 package and its extensions. Additionally, the nonparametric Mann–Whitney U-test was used to test the significance of differences in *mcyE* abundances and crustacean ingestion of *mcyE*-containing cells between sampling sites. The workflow was performed using STATISTICA 13.2 software.

## Figures and Tables

**Table 1:** Water quality parameters in sampling sites in Lake Peipsi *s.s.* and Lake Lämmijärv in 2021 and trophic state classification according to Caspers [36].

	P11 Peipsi *s.s.*		P38 Peipsi *s.s.*		P17 Lämmijärv	
	Mean	Range	Mean	Range	Mean	Range
TP, µg/L	37.83	23–58	41.17	26–78	101	39–160
TN, µg/L	706.67	380–1000	901.67	530–1300	1091.67	660–1600
NO_3_^−^, µg/L	37.17	20–100	143.83	20–610	58.83	20–220
NO_2_^−^, µg/L	3.50	3–6	4.33	3–7	3.83	3–7
NH_4_^+^, µg/L	27.17	10–53	23	10–74	24	10–44
Water Temp, °C	15.8	7–25	16.0	6.9–25.4	15.9	6.7–25.7
Secchi depth, m	1.7	0.8–2.4	1.2	0.6–1.3	0.7	0.5–1.1
pH	8.52	8.2–8.8	8.47	7.9–8.8	8.51	8.2–9.2
O_2_, mg/L	10.38	8–13.2	10.25	8.4–12.1	9.78	7.2–11.8
Chl-*a*, µg/L	17.95	6.1–30.5	22.65	6.3–44.8	57.77	20.9–109
OECD clasif.	Eutrophic		Eutrophic		Eutrophic/hypertrophic	

**Table 2:** Mean and maximum taxon-specific ingestion and mean weight-specific ingestion of potentially toxic *Microcystis* cells (based on *mcyE* synthetase gene detection in consumer’s guts) of the most abundant cladoceran and copepod taxa in Peipsi in 2021.

Taxon	Average (±SD) Length, mm	Maximum Taxon-Specific Ingestion, *mcyE* Cells/ind	Mean (±SD) Taxon-Specific Ingestion, *mcyE* Cells/ind	Mean (±SD) Weight-Specific Ingestion, *mcyE* Cells/100 mg
Cladocerans				
*Daphnia* spp.	1.34 ± 0.21	303	20 ± 21	30 ± 25
*Bosmina* spp.	0.66 ± 0.13	61	16 ± 17	50 ± 65
*Chydorus sphaericus*	0.31 ± 0.03	22	3 ± 5	216 ± 150
Copepods				
*Eudiaptomus gracilis*	1.16 ± 0.09	74	12 ± 23	60 ± 60
*Heterocope appendiculata*	1.93 ± 0.12	6428	1203 ± 2192	611 ± 1004
*Mesocyclops leuckarti*	0.85 ± 0.07	610	92 ± 138	494 ± 562

**Table 3:** Spearman correlation coefficients (r_s_) between the total crustacean population ingestion of toxigenic *Microcystis* (based on *mcyE* synthetase gene detection in grazer gut content) and various grazers’ abundance, biomass; *Microcystis* biomass; *mcyE* gene copy numbers in Lake Peipsi water in 2021.

Grazers	Total Crustacean Ingestion (*mcyE* Cells/L) vs. Grazer Abundance	Total Crustacean Ingestion (*mcyE* Cells/L) vs. Grazer Biomass	*Microcystis* Biomass vs. Grazer Biomass	*McyE water* (Copies/L) vs. Grazer Biomass
Cladocerans	0.398	0.311	−0.607 **	−0.718 **
Copepods	0.684 **	0.425	−0.318	−0.346
Crustaceans	0.684 **	0.368	−0.571 *	−0.732 **
*Daphnia* spp.	0.23	0.204	−0.674 **	−0.666 **
*Bosmina* spp.	0.01	−0.032	−0.108	−0.2803
*C. sphaericus*	0.251	0.322	0.118	0.2795
*E. gracilis*	0.610 *	0.538 *	−0.084	−0.3067
*M. leuckarti*	0.705 **	0.620 *	−0.329	−0.1787
*H. appendiculata*	−0.698	0.030	0.151	−0.2125
*L. kindtii*	−0.243	0.099	−0.361	0.0311
*B. longimanus*	−0.57	−0.059	−0.479	0.0035

Correlation is significant at * *p* < 0.05; ** *p* < 0.01

**Table 4:** Spearman correlation coefficients (r_s_) between environmental variables and cladoceran, copepod, and total crustacean population ingestion (*mcyE*-containing cells/L), *Microcystis* biomass, and *McyE* copy numbers in Lake Peipsi water in 2021.

Variables	Cladoceran, Ingestion (*mcyE* Cells/L)	Copepod, Ingestion (*mcyE* cells/L)	Total Crustacean Ingestion (*mcyE* Cells/L)	*Microcystis* (mg/L)	*McyE* Water (Copies/L)
Water temp, °C	0.443	0.288	0.461	−0.237	−0.296
NO_3_^−^_,_ µg/L	0.436	−0.346	−0.142	−0.467	−0.399
NH_4_**^+^**, µg/L	0.393	0.161	0.014	−0.244	−0.335
NO_2_^−^, µg/L	0.288	−0.026	0.159	−0.479	−0.467
TN, µg/L	0.412	−0.163	−0.225	0.321	0.607 *
TP, µg/L	−0.036	0.431	0.256	0.726 **	0.637 *
PO_4_**^+^**, µg/L	−0.246	0.358	0.211	0.888 ***	0.632 *
Secchi depth, m	0.075	−0.222	−0.052	−0.758 **	−0.571 *
pH	−0.045	0.189	0.119	0.097	−0.03
Chl-*a*, µg/L	0.016	0.463	0.293	0.814 ***	0.664 **

Correlation is significant at * *p* < 0.05, ** *p* < 0.01 and *** *p* < 0.001, respectively.

## Data Availability

The original contributions presented in this study are included in the article/Supplementary Material. Further inquiries can be directed to the corresponding author(s).

## Supplementary Materials

The following supporting information can be downloaded at https://www.mdpi.com/article/10.3390/toxins17010042/s1, Figure S1: Composition, abundance, and biomass of crustacean zooplankton communities in Lake Peipsi in 2021 in sampling sites P11, P38, and P17. Cladocerans (A, B); copepods and predatory cladocerans (C, D). Figure S2: Temporal variation in the individual ingestion of potentially toxic *Microcystis* cells based on *mcyE* synhetase gene detection in the gut contents of the studied cladoceran and copepods taxa in Peipsi (*Daphnia* spp., *Bosmina* spp., *Chydorus sphaericus*, *Eudiaptomus gracilis*, *Mesocyclops leuckarti*, *Heterocope appendiculata*, *Leptodora kindtii*, *Bhythotrephes longimanus*) in 2021. Figure S3: Temporal variation in most abundant grazer population (*Daphnia* spp., *Bosmina* spp., *Chydorus sphaericus*, *Mesocyclops leuckarti*, *Heterocope appendiculata*, *Eudiaptomus gracilis*, and cladocerans, copepods, and total crustaceans) ingestion of potentially toxic *Microcystis* cells in liters of lake water in Peipsi in 2021 based on *mcyE* synthetase gene detection in grazer gut contents.
