# Supplementary material for: Crustacean Zooplankton Ingestion of Potentially Toxic Microcystis: In Situ Estimation Using mcyE Gene Gut Content Detection in a Large Temperate Eutrophic Lake
Source: Toxins (Basel). 2025 Jan 16;17(1):42. doi: 10.3390/toxins17010042 (PMC11768910; doi:10.3390/toxins17010042)
Supplement: Supplementary file 1 [file toxins-17-00042-s001.zip › toxins-3354622-supplementary.pdf]

Supplementary Material: Crustacean Zooplankton Ingestion of Potentially Toxic *Microcystis*: In Situ Estimation Using *mcyE* Gene Gut Content Detection in a Large Temperate Eutrophic Lake

Helen Agasild, Margarita Esmeralda Gonzales Ferraz, Madli Saat, Priit Zingel, Kai Piirsoo, Kätlin Blank, Veljo Kisand, Tiina Nõges and Kristel Panksep

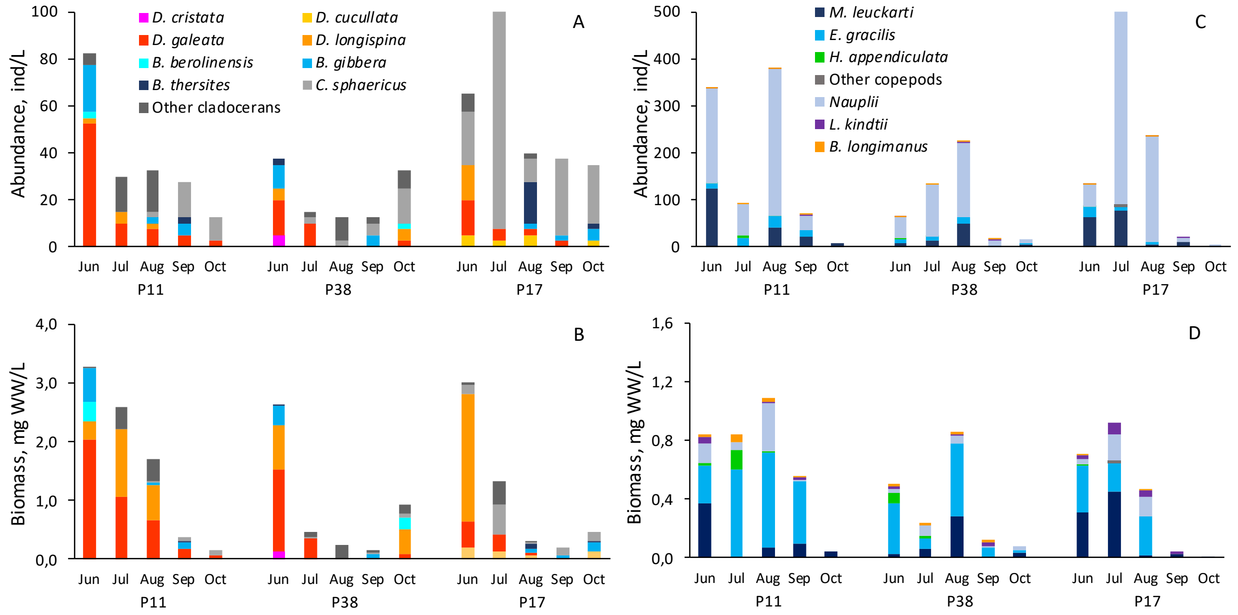

Figure S1. Composition, abundance and biomass of crustacean zooplankton communities in Lake Peipsi in 2021 in sampling sites P11, P38, P17. Cladocerans (A, B); copepods and predatory cladocerans (C, D).

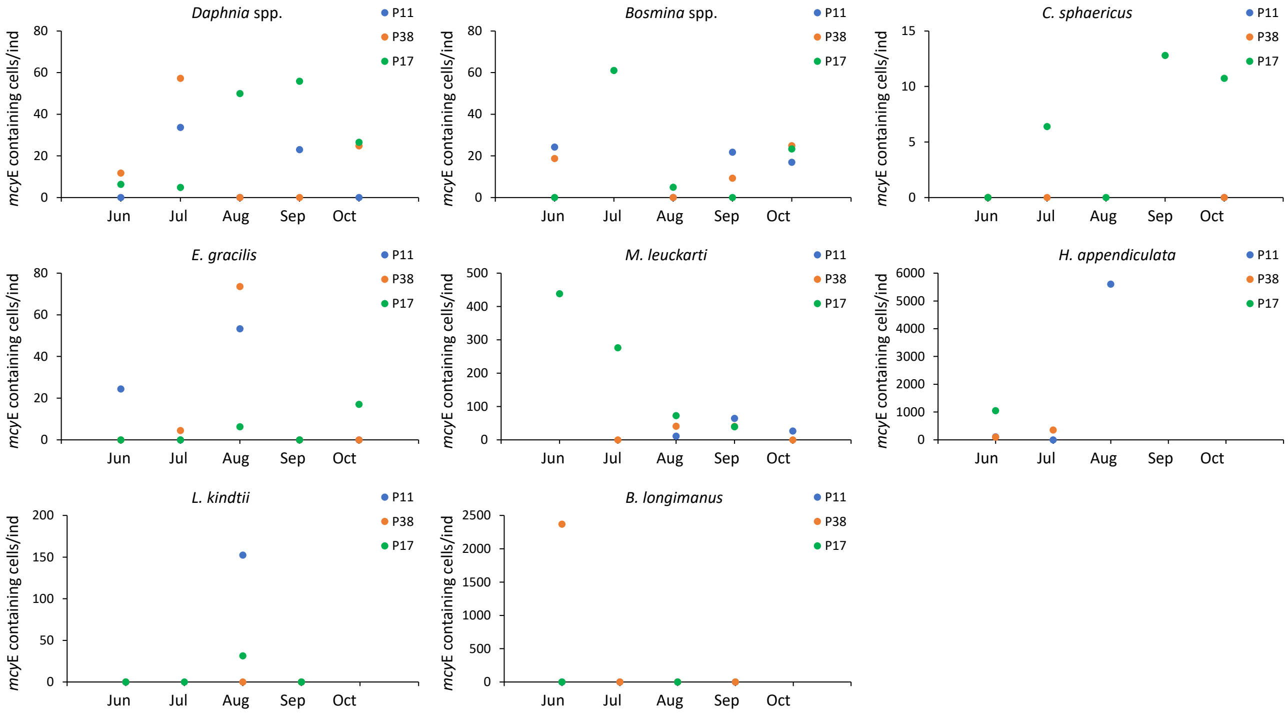

Figure S2. Temporal variation of individual ingestion of potentially toxic *Microcystis* cells based on *mcyE* synthetase genes detection in gut contents of the studied cladoceran and copepods taxa in Peipsi (*Daphnia* spp., *Bosmina* spp., *Chydorus sphaericus, Eudiaptomus gracilis, Mesocyclops leuckarti, Heterocope appendiculata, Leptodora kindtii, Bhythotrephes longimanus*) in 2021.

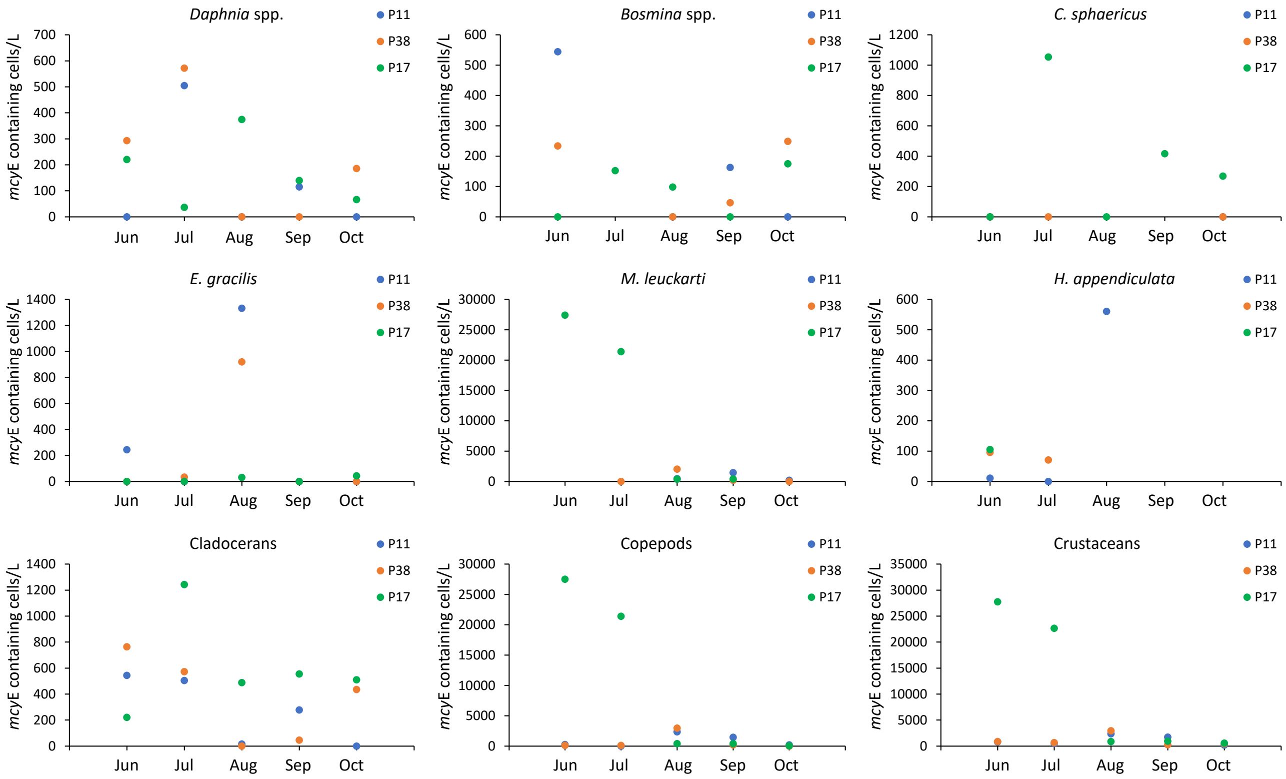

Figure S3. Temporal variation of most abundant grazers' populations (*Daphnia* spp., *Bosmina* spp., *Chydorus sphaericus, Mesocyclops leuckarti, Heterocope appendiculata, Eudiaptomus gracilis,* and cladocerans, copepods and total crustaceans) ingestion of potentially toxic *Microcystis* cells in liter of lake water in Peipsi in 2021 based on *mcyE* synthetase genes detection in grazer gut contents.
